# Supplementary material for: MetaRibo-Seq measures translation in microbiomes
Source: Nat Commun. 2020 Jun 29;11:3268. doi: 10.1038/s41467-020-17081-z (PMC7324362; doi:10.1038/s41467-020-17081-z)
Supplement: Supplementary file 10 — Supplementary Data 7 [file 41467_2020_17081_MOESM10_ESM.zip › File2/Confidence_VeryHigh_Taxonomy/122464_out.krona.html]

Javascript must be enabled to view this page.

members
magnitude
magnitudeUnassigned
count
unassigned
taxon
rank

122464\_out

30

30
superkingdom
2

1239
30
phylum

186801
class
30

order
186802
1

SRS143780\_contig\_number\_32872
30

186803
29
family

genus
27
189330


SRS011134\_contig\_number\_7038SRS013940\_contig\_number\_contig-100\_11628.11629SRS014923\_contig\_number\_29436SRS014979\_contig\_number\_1573SRS015217\_contig\_number\_28968SRS015264\_contig\_number\_contig-100\_9586.9586SRS016095\_contig\_number\_13108SRS017521\_contig\_number\_38639SRS019068\_contig\_number\_80676SRS043768\_contig\_number\_contig-100\_6931.140854SRS049712\_contig\_number\_contig-100\_3328.3328SRS050422\_contig\_number\_5407SRS050752\_contig\_number\_7967SRS051031\_contig\_number\_contig-100\_25514.112773SRS052697\_contig\_number\_10291SRS056259\_contig\_number\_contig-100\_9498.44527SRS077502\_contig\_number\_contig-100\_3618.107214SRS098571\_contig\_number\_contig-100\_18892.198326SRS098644\_contig\_number\_8275SRS143148\_contig\_number\_16538SRS143342\_contig\_number\_contig-100\_1929.43441SRS143876\_contig\_number\_1977SRS146812\_contig\_number\_12123SRS147022\_contig\_number\_10263SRS148159\_contig\_number\_contig-100\_522.103965SRS148874\_contig\_number\_5395SRS149879\_contig\_number\_22170
27
species
39486

species

SRS097889\_contig\_number\_38604
1
1200721

841
genus
1

1262942

SRS012969\_contig\_number\_contig-100\_2384.129212
1
species
